# "Malignant" mitral stenosis

**DOI:** 10.1186/1749-8090-7-19

**Published:** 2012-03-08

**Authors:** Johann Auer, Robert Berent, Franz Gurtner

**Affiliations:** 1 Department of Cardiology and Intensive Care, General Hospital Braunau, Braunau, Austria; 2 Department of Cardiology and Intensive Care, General Hospital Simbach, Simbach, Germany; 3 Center of Cardiac Rehabilitation, Bad Schallerbach, Austria

**Keywords:** Metastasis, Heart failure, Dyspnea, Echocardiography, Computed tomography

## Abstract

Symptomatic mitral stenosis caused by a left atrial mass as the first sign of metastasis of a malignant tumor is extremely rare and frequently associated with poor prognosis. We report a case of a 59-year-old man with a history of grade 3 malignant fibrous histiocytoma on his left tigh treated by limb-sparing surgery 17 months earlier, who was admitted with 10-days of worsening dyspnea. Imaging revealed a left atrial mass protruding through the mitral valve that resulted in severe mitral stenosis. Biopsy confirmed metastasis of malignant fibrous histiocytoma.

## Background

Symptomatic mitral stenosis caused by a left atrial mass as the first sign of metastasis of a malignant tumor is extremely rare and frequently associated with poor prognosis. Atrial tumours presenting as mitral stenosis are most commonly myxomata, occasionally pedunculated sarcoma, and very rarely metastases.

## Case Presentation

**Figure 1:**
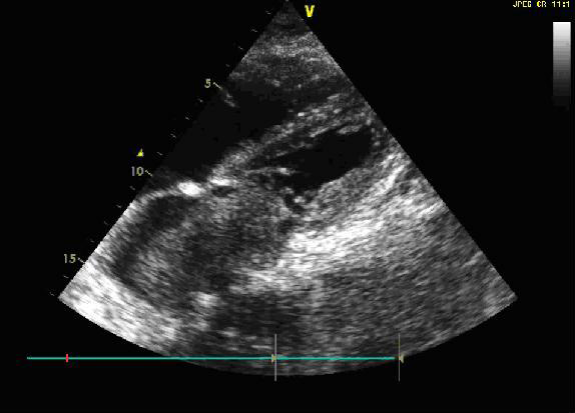
**Echocardiography shows a giant left atrial mass**.

**Figure 2:**
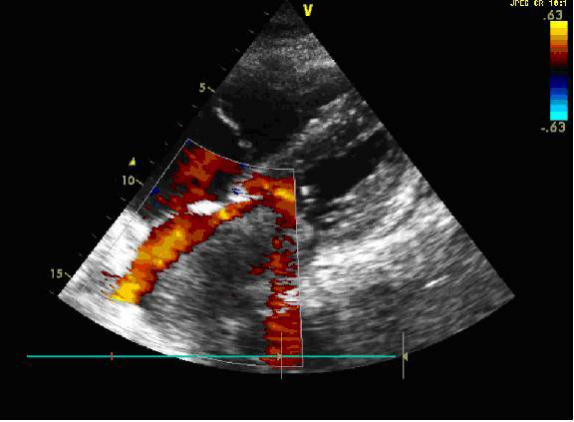
**Echocardiogram with a left atrial mass protruding through the mitral valve**.

**Figure 3:**
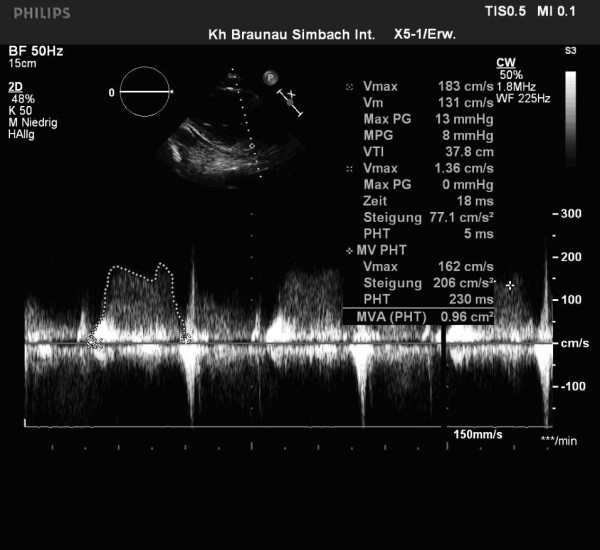
**Continous wave (CW) - spectral Doppler tracing indicating mitral stenosis with a mitral valve area less than 1.0 cm2**.

**Figure 4:**
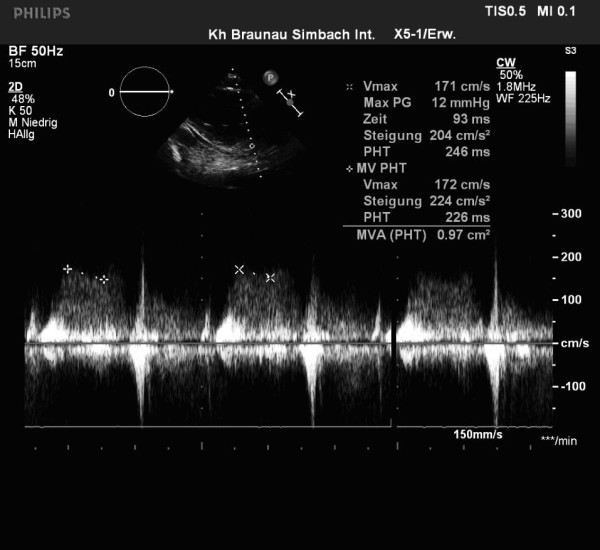
**CW - spectral Doppler tracing indicating severe mitral stenosis**.

**Figure 5:**
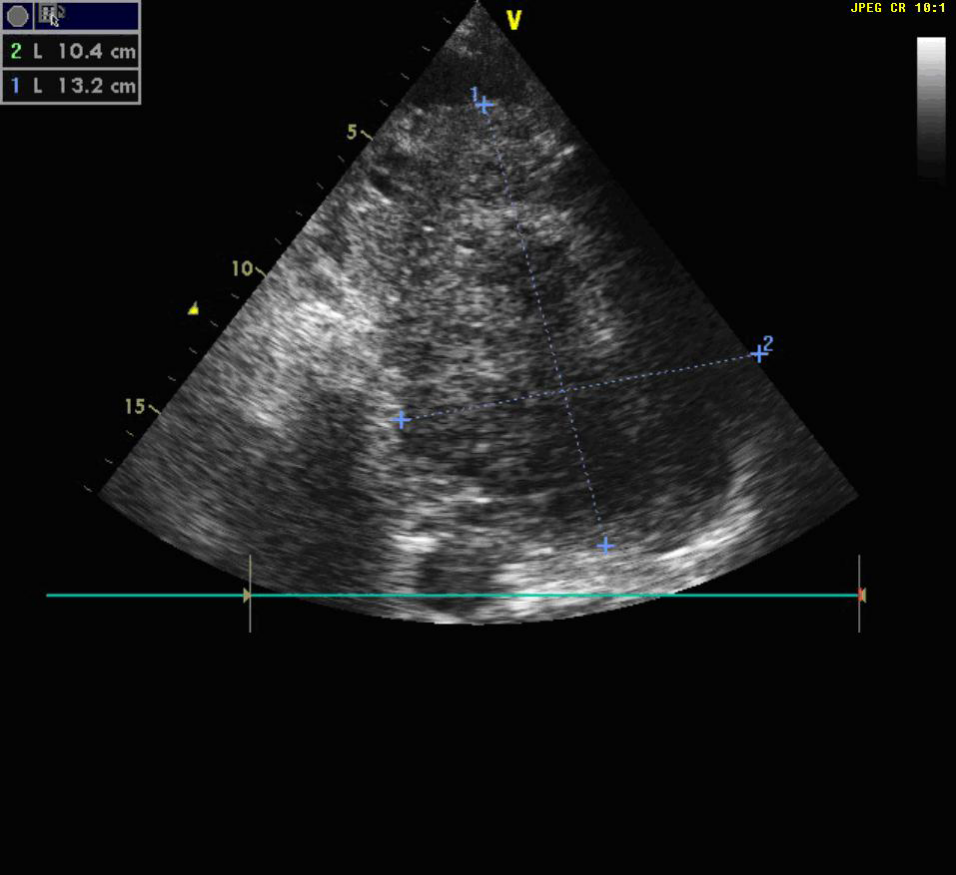
**Sonography demonstrating a giant mass in the left pleural space penetrating the left pulmonary veins**.

**Figure 6:**
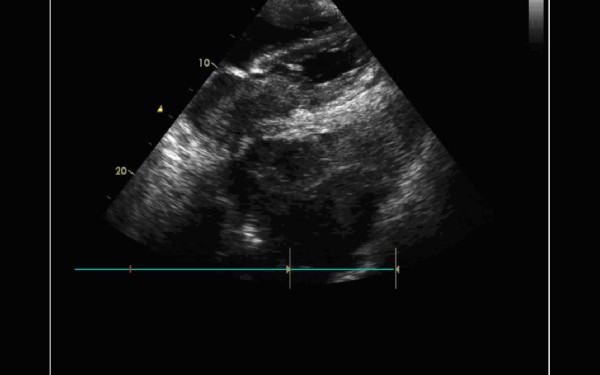
**Sonography with a large mass in the left pleural space**.

**Figure 7:**
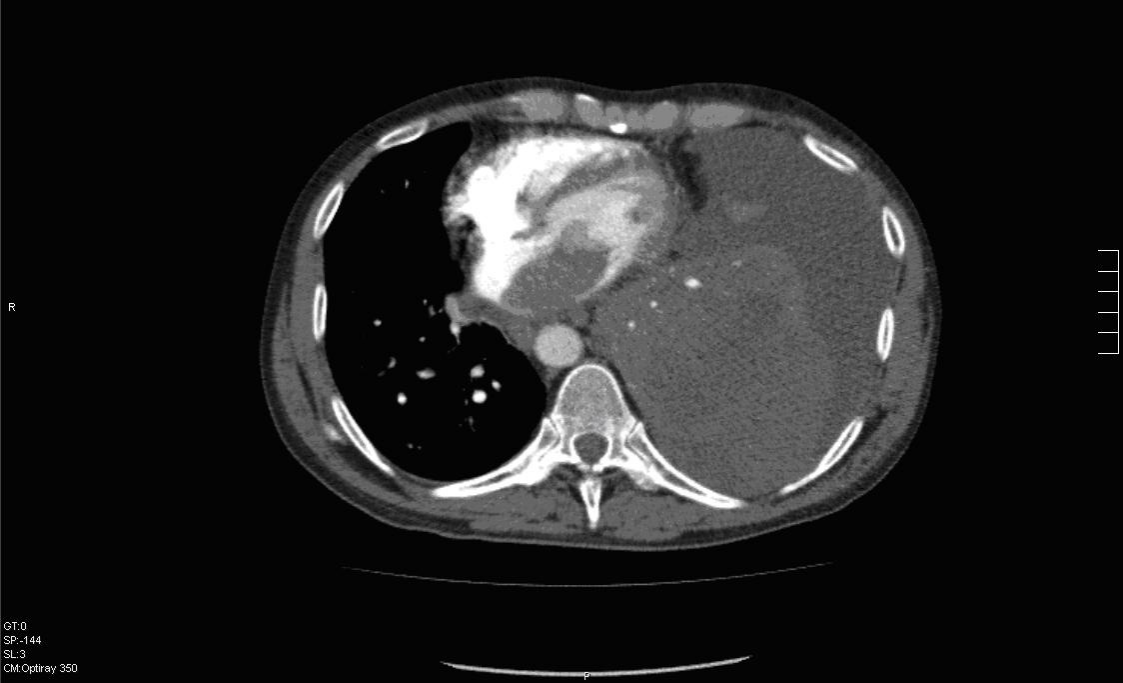
**Computed tomography shows a large mass in the left pleural space**.

**Figure 8:**
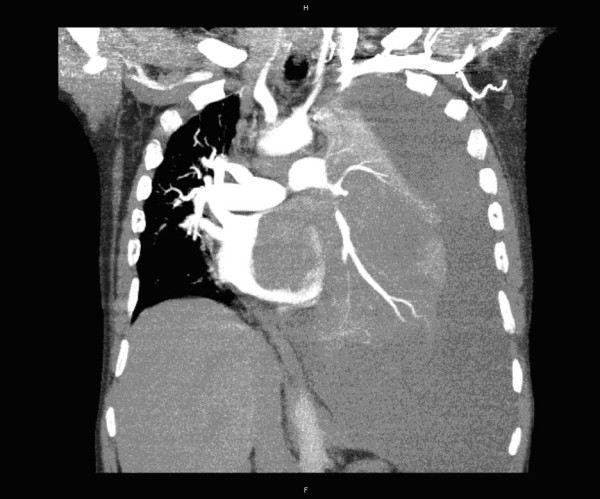
**Computed tomography shows a large mass in the left pleural space penetrating the left pulmonary veins and protruding to the left atrium and through the mitral valve**.

**Figure 9:**
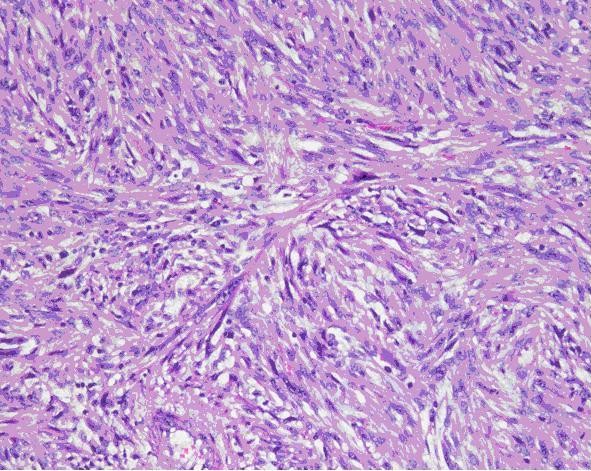
**Biopsy of the pleural tumor revealed metastasis of malignant fibrous histiocytoma**.

A 59-year-old man with a history of grade 3 malignant fibrous histiocytoma on his left tigh Stage IIA (pT1bN0M0) treated by limb-sparing surgery 17 months earlier, was admitted with 10-days of worsening dyspnea. The patient underwent postoperative chemotherapy after surgery and had follow up visits every six months. Blood pressure and heart rate were 150/85 mmHg and 136 beats/minute, respectively. Cardiac auscultation revealed a diastolic murmur. End-inspiratory crackles suggested pulmonary edema. Echocardiography revealed a left atrial mass protruding through the mitral valve (Figures 1 and 2). Continous wave spectral Doppler showed mitral stenosis with a mitral valve area less than 1.0 cm^2 ^(Figures 3 and 4). Additionally, a giant mass in the left pleural space penetrating the left pulmonary veins could be demonstrated by ultrasound (Figures 5 and 6) and by computed tomography (Figures 7 and 8). Biopsy of the pleural tumor revealed metastasis of malignant fibrous histiocytoma (Figure 9). Pulmonary edema resolved with symptomatic treatment. Before discussing further treatment options, the patient died suddenly four days after admission.

## Conclusion

Atrial tumours presenting as mitral stenosis are most commonly myxomata, occasionally pedunculated sarcoma, and very rarely metastases [1,2].

Symptomatic mitral stenosis caused by a left atrial mass as the first sign of metastasis of a malignant tumor is extremely rare and frequently associated with poor prognosis [1-3]. However, there are some reports about successful favourable response with combined treatment particularly in patients with high tumor mitotic rate [4,5].

## Consent

Written informed consent was obtained from the patient for publication of this report and any accompanying images.

## Competing interests

The authors declare that they have no competing interests.

## Authors' contributions

JA wrote the manuscript and formatted the images. FG provided cardiovascular images and reports. RB supervised and revised the draft manuscript. All authors read and approved the final manuscript.
